# A Happy Christmas

**Published:** 1868-12

**Authors:** 


### A HAPPY CHRISTMAS

Is an impossibility to any man who has any money to pay within twenty days of New Year. This is a kind of negative "*recipe*," reader, but if you will attend to it, there will be a quietude and a luxurious "abandon," about Christmas Eve, which few people ever know. For the sake of humanity, try it once—only once—and the beauty of it will so enrapture you that it will never be forgotten. So, go and pay every cent you can, the moment you lay down this journal; and if there is a demand which you cannot pay, make a *timely* and *satisfactory* arrangement, so that your creditor may not calculate on you. He'll think the better of you for it, and be easier with you hereafter. This thing of waiting until the very last moment, and then going to a man who was relying on you, with your finger in your mouth, and a hang-dog look, is enough to give an honorable man an apoplexy.

